# Understanding Learners’ Metacognition of Online Teacher Feedback Amid COVID-19: A Case Study in a University Livestream Instruction Context

**DOI:** 10.3389/fpsyg.2022.861845

**Published:** 2022-04-27

**Authors:** Feng Wang, Fanding Meng, Shichuan Liu, Shiqi Wang, Lihui Pan, Zhong Lin

**Affiliations:** ^1^School of Foreign Languages, Xidian University, Xi’an, China; ^2^School of Foreign Languages, Chang’an University, Xi’an, China; ^3^School of Foreign Languages, Sichuan University of Arts and Science, Dazhou, China; ^4^School of Foreign Languages, East China Normal University, Shanghai, China; ^5^School of Liberal Arts, Guangxi University, Nanning, China; ^6^School of Foreign Languages, Chang’an University, Xi’an, China

**Keywords:** metacognition, feedback, online L2 education, post-pandemic era, COVID-19

## Abstract

While research on metacognition in second language (L2) learning has burgeoned in the past two decades, its relation to actual teaching behaviors, such as teacher feedback, remains to be fully described and explained in L2 classroom, especially in livestream English teaching settings. To fill this gap, this case study examined how learners utilize and regulate metacognition of online teacher feedback during COVID-19 in a Chinese inner land university. Data were gathered through semi-structured interviews. With qualitative and interpretive analysis, it is revealed that leaners positively receive online teacher feedback for its detrimentalness together with a growth mindset and high levels of resilience, but, on the whole, there is a metacognitive deficit: they misinterpret self-consciousness about online feedback which is underpinned by a conception of tasks that characterizes online L2 learning. This research expands our understanding of L2 learning processes pertaining to awareness and management of teacher feedback receiving and may also shed light on solutions to empower livestream teaching by building external scaffolding devices to compensate weaknesses of online L2 education during the pandemic and beyond.

## Introduction

Since January 2020, the COVID-19 has been raging all over the world and caught countries off guard, exerting a huge impact on all walks of life. This outbreak has undoubtedly posed unexpected challenges to the field of education so that traditional teaching methods, amid the epidemic, cannot function normally, bringing about new complications for language instruction, and learning. Almost all education institutions in China closed campuses and have quickly shifted to online instruction. Therefore, livestream education plays a great part in supporting regular education, but the abrupt alteration from the traditional offline model undoubtedly challenges both learners and instructors. On the one hand, without face-to-face communication and surveillance from teachers as usual in classroom, learners have to be able to learn autonomously with good self-disciplinedness and reflect regularly on their own learning rate and attainment. On the other, of much necessity for teachers is to know what the learners are weak at with their uncontrolled learning at home. Consequently, one emerging issue goes to the way of providing and receiving high-caliber teacher feedback in an unfamiliar teaching environment.

Teacher feedback, both a medium to present learners with messages vis-à-vis their performances and an adjusting mode of the learning processes (Chou and Zou, 2020), has long been documented as an important bridge connecting both teachers and learners to facilitate instruction and learning. However, traditional practices of teacher feedback giving fall short of the new situation, so that teachers have to change their feedback practice for the virtual online environment (Jiang and Yu, 2021). Likewise, students have to adapt when they receive online teacher feedback. In effect, language learners deploy varied metacognitive, among other learning strategies, to fulfill their learning tasks.

Although recent studies have touched upon interventions for development of metacognition, methods of improving metacognitive, and metacognitive calibration in online learning (Saenz et al., 2019; O’Loughlin and Griffith, 2020; Zhao and Ye, 2020), awareness and management of learner metacognition of online teacher feedback receiving is still found lacking in extant research on second/foreign language learning psychology. Therefore, this research, broadly subsumed under what is known as leaner psychology, aimed to fill this gap by exploring learners’ practice of metacognition, zooming on metacognitive awareness and regulations when and after they receive online teacher feedback for their online learning task during COVID-19.

## Literature Review

To filter out related and representative literature, the author searched online databases of ProQuest, ERIC and Google Scholar with the combinations of the key terms: Feedback, metacognition, online learning, and foreign language. A plethora of research within area of language learning has attempted to elucidate the role of different language skills, and a number of investigations have conducted to foster metacognition in the classroom, but as far as the context of online distance instruction in the field of second language education is concerned, the literature is limited. Accordingly, the online learning during the COVID-19 in second language education sets the context for this study.

### Online Learning and Teacher Feedback in Online Environment

Online learning is alternatively termed as online education (Harasim, 1989), E-learning (Charp, 2001), blended-learning (Ryan et al., 2016), web-based education (Khan, 1997), among many others. Research has attempted to unscramble the interpretations of the concept completely and systematically. Curtain (2002) conceptualizes online learning as “the application of the internet in several ways to establish the interaction between teachers and students.” From the perspective of learners, Singh and Thurman (2019) categorized online learning as “learning experiences in synchronous or asynchronous conditions with the help of different mediums to access, in which learners can learn from anywhere, anytime, in any rhythm.” Both these understandings underpin the reliance of learning on modern advances of the internet and computer science. For this study, online learning is referred to as the synchronous learning activities of lectures, forums, and interactions by teachers and students after class via online interactive software, which serves as a complementary method to traditional classroom face-to-face instruction.

To improve learning and compensate the insufficiency and inconvenience of interaction in online environment, teacher feedback works as one of the crucial guarantees for effective and efficient learning (Ramaprasad, 1983; Mory, 2004; Parkin et al., 2012; O’Donovan et al., 2021). Generally, feedback can be conducted in diverse forms, but in class it frequently occurs in formative form through which teachers are able to learn about students’ learning and the students are able to learn about their own performance, before determining what to do in next steps (Black and Wiliam, 2009). In line with Hattie and Timperley (2007) who conclude that feedback, as one of the most powerful element, can influence learners’ learning in a wide range of educational conditions, teacher feedback is considered powerful to develop second language learners’ metacognition and support their online learning as well. Previous research has already discussed some key advantages of feedback. For instance, Eppich et al. (2015) address that high-quality feedback was conducive to competency development; Duijnhouwer et al. (2012) illustrate feedback as information was the medium to improve learning achievements through adjusting learner’s awareness, cognition and behavior; Narciss et al. (2014) pinpoints two superiorities of feedback in helping learners recognize the disparities between the ideal and existing condition and supporting learners in acquiring learning methods and regulating learning process; Nicol and Macfarlane-Dick (2006) regards feedback as an instrument that could motivate learners. These standpoints were carefully drawn from systematic analysis, rather than roughly generalized with impulses, through which the positive effects of online feedback during the COVID-19 as the complementary of hard-copy feedback are obviously demonstrated.

Based on Ambler et al. (2014) and Hast and Healy (2018) hold that feedback enables repeated access of feedback and that the characteristic of privacy is also a big plus, in which online feedback could “relieve the inferiority of the learner whose academic record is relatively low.” Considering these virtues, learners and staffs all share partiality for online feedback over traditional hard-copy feedback. Therefore, the application of online feedback can serve as a reference for future research and a benefit for learning.

However, challenges for effective feedback still exist. In the context of online environment, it is tough for learners to control the real situation of their own due to the difficulty with face-to-face communication (Zhang and Zhu, 2018; Louis-Jean and Cenat, 2020) and the absence of learning guidance in the process of learning. Hence, the barriers specific to online feedback must be taken into consideration seriously. Mensink and King (2020) investigated the level of concern with online feedback. The lack of timely explanation and clarification for online feedback may “lead to misunderstanding and difficulties” (Hattie and Timperley, 2007). Moreover, along with the renovation of web technologies, there emerges a new round of huge development in the internet industry which requires an extra commitment of time and energy by both instructors and learners to learning about the various functions of the online software.

Due to pandemic, online learning has turned into a hot issue of much greater concern than ever, and recent studies have been undertaken from varied aspects (e.g., Joosten and Cusatis, 2020; Mensink and King, 2020; Hergüner et al., 2021; Wang et al., 2021). Researchers agreed on the fact that many students enrolled in online education and adapted gradually to the new learning environment, their experiences vary from person to person. What’s more, the absence of learner engagement with feedback has been recognized as a long-standing problem over the years (Hyland, 2003; Handley et al., 2011). Hence, this study, sets it special focus, in view of this, on teachers’ feedback in relation to learners’ metacognition in the online learning space under the COVID-19 circumstances.

### Feedback and Metacognition

As teacher feedback is substantial to the learning process and in turn influences learning adjusting and monitoring, investigations (e.g., Callender et al., 2016) have been carried out to develop metacognition of teacher feedback with the eventual objective of enhancing learning achievements (see Drigas and Mitsea, 2021). Flavell (1979), an American psychologist, introduced metacognition as “cognition of cognition” or “thinking about thinking,” meaning the cognition or recognition of one’s own cognitive activities, which can be applied in different fields. Although interpretations of the concept of metacognition in education are diverse and multidimensional, they are all formulated around a common core-learners’ self-consciousness, monitoring and adjustment of their own learning processes (e.g., Livingston, 2003; Martinez, 2006; Dunlosky and Metcalfe, 2008; Rhodes, 2019; Padmanabha, 2020). What’s more, scholars also come up with their own theoretical frameworks (e.g., Flavell, 1979; Hacker et al., 1998; Hattie and Timperley, 2007; Drigas and Mitsea, 2020, 2021). Among them the most relevant and applicable to this research is the theory by Hattie and Timperley’s (2007), of which the highlighting part elucidates that “…Feedback at the aspect of metacognition concentrates on developing learners’ ability of self-awareness, which can be achieved in many ways, such as encouraging learners to continue learning by affecting their self-efficacy, self-beliefs and self-regulation (Hattie and Timperley, 2007).” As is consistently approved by educational psychologists (Schraw, 2001), metacognition is composed of (1) metacognitive knowledges of self, strategies and tasks and (2) metacognitive regulation, including metacognitive monitoring and metacognitive control. This theoretical understanding informed this present research.

Evidence that proved feedback a significant way to cultivate metacognition is mixed (Poulos and Mahony, 2008; Lee et al., 2015). Due to the differences of feedback in terms of its form or content, researchers have come to different conclusions. For instance, Azevedo and Hadwin (2005) pointed out that too frequent immediate feedback would make learners feel dependent, it being not a conduction to learners’ thinking but a hinder to the cultivation of learners’ metacognition, whereas online methods associated with feedback are often presented in a positive perspective, viz., online feedback is held favorably for it helps faster marking and reducing the pressure on the faculty. In addition, online feedback is regarded as valid as feedback in hard copy (Parkin et al., 2012), so much so that it is supposed to be more well-directed and more available in the online environment.

There is also some evidence that feedback has a positive effect on self-regulated learning. Black and Wiliam (2009) held that formative feedback could be regarded as the effective means to make up the learning gaps and improve learners’ metacognition. Callender et al. (2016) also provided overwhelming evidence of the positive relationship between feedback and metacognitive accuracy, declaring that adequately “powerful” individual feedback could develop metacognition.

### Research Gaps and Questions

Although research has elaborated the tenets of metacognition to teacher feedback, the practice and management of metacognitive of online L2 teacher feedback is still in its infancy, poorly understood and rarely explored. In addition, several studies have also indicated that part of the learners showed dissatisfaction with the received feedback in the online context (Mulliner and Tucker, 2017; Hast, 2021). Therefore, it is important to inquire how feedback is exactly drawn on in the online context as it assists to determine the anxiety-alleviating strategies for online learners. In view of the traced research gap above, this research conducts an exploratory study of second language learners’ metacognition of feedback that significantly underpins their online second language learning outcomes. It will seek to address the following research questions:

(1) How do second language learners utilize and regulate metacognition when receiving online feedback?
(2) What factors contribute to language learners’ experience of their metacognition of online feedback?

## Methodology

In order to develop an understanding of online feedback in terms of metacognition, this research conducted a multi-case study through a qualitative method with focus on a small sample of English majors for an in-depth investigation. This method is considered as effective to provide great help for teachers and students both in cognitive tools and learning environment, and enrich learning activities in the online environment.

### Research Approach

Case study is defined by Gerring (2004) as “an in-depth study of a single unit with a target to generalize across a larger set of units.” It is applicable to this research since it deals with the specific phenomenon of metacognition practice in online feedback as a single case which will be analyzed and described in detail. The interview is regarded as one of the momentous instruments and the most frequently used methods within a case study by scholars for its natural and acceptable way of accumulating messages.

On the basis of the above analytical approaches and in line with the descriptions about feedback and metacognition, this research was in progress with students of English majors in a Chinese inner land university and unfolded against a backdrop of online education during the COVID-19.

### Research Instrument

As mentioned earlier, the interview is regarded as a common medium to explore metacognition in second language education by many scholars. Before developing the interview guideline, we determined the interview’s structure, degree of formality, contact mode, frequency, form and number of the interviewees. To control the structure of the interview and allow the respondents to participate actively, the semi-structured interview was adopted in this research, that is, an interview guideline was drawn up in advance with questions being asked to the respondents correspondingly. The method of one-time interview was adopted mainly to collect factual information. As to the form, a formal and direct interview was handled in a face-to-face conversation between researchers and participants on a certain issue at a predetermined time and place. In selection of our interview form, the research questions, objectives and participants were taken into consideration. In this research, individual interviews were directed to deal with the specific questions of metacognition conceptualization in online feedback. Grossly, full preparations for interview served to ensure the smooth progress of the research and boost the quality of the interview, including designing of the interview guideline, selecting of the interviewee, choosing of time and place and relationship establishment, etc. Next, the design of the interview outline will be discussed.

By following the research objectives and tracking the literature related to metacognition and feedback, this research resolved which types of data need to be collected. The interview guideline for this research consists of two parts: First are metacognition and its composition in terms of online feedback, i.e., metacognitive knowledge of self, of strategies and of tasks, and metacognitive regulation composed of metacognitive monitoring and metacognitive control; second is a list of 6 questions corresponding to the three research questions. The interview referenced Schraw and Moshman’s (1995) work in line with the belief that “Metacognitive knowledge is about the knowledge of the cognitive subject,” which is, in this research, manifested as the characteristics and status of the learners, that is, learners understand their own abilities when learning new knowledge, know the privileged learning strategies that are instrumental to their learning, and comprehend which concrete task environment is of avail. In this way, it is imperative to elaborate a worthy mode to design the question on learners’ experiences of online feedback such as their individual attitudes when receiving feedback, their degree of acceptance and interpretation of online feedback, and identify the types, forms and contents of the online feedback received by the learners in the online environment during the COVID-19. Furthermore, Nelson and Narens’ (1990) and Schraw’s (2001) standpoints on metacognitive regulation supply a deep-going aspect of metacognition in online feedback in this present research. As far as this research is concerned, metacognition regulation is represented as learners monitoring and controlling their learning process after interpreting the online feedback. Under such circumstances, the investigation on how the participants dealt with or intended to do with feedback and whether they revise it or not after receiving feedback are also embodied in the interview guideline. Last, based on Hattie and Timperley (2007), the research invited participants who were asked about how helpful they found feedback for metacognition and encouraged to describe some strategies that might support their metacognition when using feedback.

### Research Participants

The interviewees were selected by basing on the sampling principle of typical case. To be specific, this research selected those who belong to the representative cases in order to understand metacognition practice in online feedback. The present study of typical cases is not to infer the results to the population sampled, but to explain what a typical case looks like in this kind of phenomenon. Furthermore, owing to the small number of subjects in the case study, those who participated must be willing to cooperate and take a serious attitude. With this, the 4 participants chosen in this research are all class monitors, two with good academic performance and the other two with average performance based on their previous GPA. The reason why monitor is chosen in the interview is that monitor, working as an aide to the teacher, is in a position to get some information at his/her disposal and thus serve typical sample in the interview. At the same time, achievement discrepancy between the participants was considered in favor of making comparison between the opinions of the participants at different achievement levels.

Conclusively, the participants in this research were 4 undergraduates of foreign language and literature from Grade 2019 in a university of Xi’an City, Shaanxi Province in the northwestern Inner Land of China. They were enrolled in online learning during the COVID-19 (the second semester in 2019–2020 academic year) to ensure that they had enough experience in receiving online feedback.

### Data Collection and Analysis

It is extremely significant to clarify the purposes of interview in order to reduce blindness and utilize the method correctly in the practical research. For this, the formal interview commenced with a brief inauguration through mutual introductions, conversations, and then was followed with a semi-structured interview.

Interviews were conducted through WeChat at participants’ convenience to ensure the smooth progress. Each semi-structured interview lasted about 30 min and was recorded and transcribed in detail. Chinese language was used as the medium of communication during the interview in order to facilitate more fluently the discussion and gain an all-round and in-depth insight into the responses the participants made in the interview.

To find key information related to the research objective mentioned by the participants, the transcripts, viz.,the interview data, were analyzed by the first researchers with the method of content analysis by generalizing and summarizing mainly through organizing notes, identifying codes and categorizing recurring parts in the participants’ responses. Disagreements were resolved through discussion and negotiation until a consensus was reached. To ensure the reliability of analysis, a third analyst, with great expertise in SLA, analyzed one fourth of the studies independently of the researchers. There was 98% agreement between the two groups.

### Findings

Findings concerning the feedback received by learners in the online learning are presented in terms of the five central themes related to metacognition and can be determined in the interview data. These fundamental themes are to present the prominent properties of the learners’ metacognition that they engendered in online feedback.

### Experiences in Receiving Online Feedback

In the interview, participants provided enough information about their experience of feedback received in the online environment, in which learners’ metacognitive knowledge of self, of strategies and of tasks can be reflected.

### Knowledge of Self in Online Feedback

According to Zhao and Ye’s (2020) and O’Donovan et al.’s (2021), learners’ metacognitive knowledge of self is generally with regard to traits or states of the second language learners in understanding their own abilities when receiving online feedback. The degree of acceptance of the online feedback and its interpretation, and the factors that may influence their online feedback, are also reflected in this aspect.

When asked about the purposes of the online feedback, participants showed their positive attitudes and all commented that the feedback, with its supervising function, could guide learners to learn more autonomously as it is with the purpose of offline feedback, as Example 1 reported:

*“Online feedback is very necessary during the epidemic. For teachers, online feedback can enable them to intuitively know the current learning situation of students and make plans for their courses that suit the needs of the students. For students, online feedback cannot only make them understand the shortcomings in the learning process, but also get critical appraisals to promote their future learning.”*

However, a general feeling was that participants’ self-cognition about themselves in receiving online feedback was below the expected standard, which corresponds to Weaver (2006) who identifies two problems with feedback, of which one highlights that learners did not have adequate comprehension of the feedback.

Example 2 demonstrated a lack of interaction that may influence their interpretation of online feedback:

*“Although online feedback is convenient and intuitive, there are some communication problems. For example, students cannot understand the meaning between the lines but only to analyze their shortcomings and correct them through a few words.”*

Example 3 also mentioned:

*“Compared with offline feedback, online feedback cannot carry out turn taking, which is detrimental for students to modify their learning.”*

Learners’ acceptance of the online feedback is widely ranged, as Examples 4 and 5 reviewed:

Example 4:

“*The COVID-19 as a special context has made it a necessity for higher education to go online. Online feedback, as an effective instrument, builds a bridge between teachers and students.”*

Example 5:

*“There is a convenience at access and submission as far as the sending and receiving of the feedback in the online environment is concerned.”*

Examples 1–5 indicate an appreciation of the characteristics of online feedback as being accessible remotely and repeatable.

In a word, the above discussions about learners’ knowledge of self-evince their awareness and approval of online feedback. However, participants’ self-cognition in receiving online feedback is still necessary to improve and the factors influencing the interpretation of online feedback deserves careful consideration. In addition, the above comments highlight the importance of training students to be proactive receivers of feedback.

### Knowledge of Strategies in Online Feedback

Knowledge of strategies hints learners’ metacognitive awareness of the application of online feedback. In other words, it refers to learners’ grasp of various ways of applying these strategies in different contexts (e.g., to set learning objectives, make learning plans, allocate learning time and choose learning environment).

In order to be more effective, the online feedback must be applied before the teacher and participants discussed how it was utilized online. Based on the participants’ comments, time arrangement seems to be the most important element to be considered among various strategies. Online feedback saves participants’ time as well as costs such as printing of assignments, as Example 2 stressed:

*“The availability of more time saved from online feedback means I can engage more carefully with the online learning.”*

In general, online feedback can directly influence the selection and application of knowledge of strategies by learners.

### Knowledge of Tasks in Online Feedback

Knowledge of tasks refers to what students know about the nature of online feedback and how they understand the requirements that online feedback sets on them, which can emerge from the interview contents.

Participants reported that online feedback changes with tasks, a response in line with Mascha and Smedley’ (2007) that “for tasks with different complexity, the quantity of feedback affects the generated effects in a different way. For complex tasks, giving some but not too much feedback is helpful. In contrast, for non-complex tasks, giving more feedback is helpful.”

Participants’ comments revealed in the application of feedback the impeding factors, including their difficulties with deciphering terminology and their unwillingness to spare effort. It is argued that these processes should be considered when organizing feedback to encourage students’ engagement with online learning.

### Metacognitive Regulation in Online Feedback

To overcome the challenges in giving appropriate feedback that can develop learners’ metacognition, delivered feedback is considered to be a significant potential as it is not optimally exploited currently. Metacognitive regulation refers to the metacognitive actions or activities, including learners’ application of their metacognitive knowledge and techniques to manage their learning process. It is often stated that metacognitive regulation consists of metacognitive monitoring and metacognitive control. Of note is that previous studies argue that the two components can play a mutually independent role (O’Leary and Sloutsky, 2019).

### Metacognitive Monitoring in Online Feedback

Previous studies showed that participants’ learning stopped with reception of teacher feedback, which may result from the fact that learners unscramble the term “feedback” superficially; only to concentrate on the achievements they have performed without being able to interpret teachers’ evaluations for development of or contribution to their study further. In this way, it is meaningful to look into the treatment of feedback received in the online environment. Participants’ comments showed their performance of metacognitive monitoring.

Example 1: *“After receiving online feedback, I was habitually concerned more about the scores of the tasks.”*

Example 2: *“I occasionally check my mistakes against the teacher’s comments.”*

These statements exhibited the salient characteristics of learners’ metacognitive monitoring emerging in their online feedback. Concerns about grades or achievements showed that students focused on learning performance at the surface level rather than explore further the learning strategies or learning methods, indicating that the learners’ major concerns, when receiving the feedback, were not with what it was about their strength and weakness in learning but with what grading it told them.

### Metacognitive Control in Online Feedback

As for metacognitive control, participants reported their revision after interpreting online feedback. Not surprisingly, all participants claimed that they would adjust their learning methods or focus on some aspects of their performance on homework in observance of the teacher feedback, aiming to improve learning efficiency and achieve better online learning effect, as Example 2 stated:

*“I will adjust learning according to the teacher feedback. If the feedback is about the learning progress, I will preview in advance before class, review in time after class, and try to keep up with the progress of the subject. If the feedback is about learning attitude, I will communicate closely with the teacher to determine what I should do in the future.*”

The above imply that learners expected their performance to be accordingly tuned-up. Although learners may recognize that their performances fall short of the requirements set by the teacher, they still have positive attitude toward their learning and communicate closely with their teachers to determine what they should do in the future. For these reasons, the following sections are conducted to help learners take control of their learning more autonomously, and at the same time, assist teachers to consider more comprehensively about feedback as an adjusting and monitoring instrument. In addition, how to organize feedback in a rational way is also discussed.

## Discussion

The findings above serve us a notification of learners’ experiences of feedback received in the online environment during the COVID-19, and demonstrate the extent they treated or revised after interpreting feedback. The findings on students’ metacognitive knowledge deficiencies have been reported in previous studies (O’Loughlin and Griffith, 2020) and can explicate their deviating attitudes toward online feedback in the specific context. For instance, Sawdon and Finn (2014) pinpoint that the majority of undergraduate students do not have metacognitive consciousness, indicating that they are not able to correctly evaluate or make appropriate revisions for their learning. In this research, it is authenticated that some undergraduates have a homologous lack of metacognitive knowledge.

Interestingly, in terms of participants’ application of online feedback, only time arrangement was discussed, showing that they were short of metacognitive awareness of strategies, which is one of the major problems that all online L2 learners regularly confront. Tanner (2012) claims that most L2 learners may have no clear understanding about metacognitive strategies before these strategies are brought into class explicitly and actively. So it seems necessary to develop learners’ metacognitive knowledge of strategies. Chan and Lam (2010) suggests formative feedback foster learners’ technique in self-evaluation and allow them to develop a series of available learning strategies. It is hence significant to instruct metacognitive strategies to learners so that they can construct learning in a self-regulated way. Saenz et al. (2019) also hold that fitting metacognitive strategies into class helps students monitor and revise their learning as well as assists them progress from novice to expert learners to some extent.

The negative factors for developing knowledge of tasks that constrain students from fluent online feedback interpreting involve the difficulty with terminology interpretation and effort expansion from participants’ interview accounts. Therefore, a number of investigations have attempted to improve knowledge of tasks in the classroom with the ultimate goal of improving student learning performance. As for metacognition in online teacher feedback, strategies that guide learners to use self-reflecting feedback encompass: (1) integrating learners’ self-regulation into feedback, including what methods learners used and how their methods were checked to be reasonable or time-consuming; (2) instructing learners to share with each other descriptive feedback on their task related to published standards, which can provide multiple perspectives to students’ metacognition in online feedback.

However, students’ capability of metacognitive regulation suggested that they appeared to be “a responsible learner” after receiving online feedback. According to Veenman (2011), it could be described as a procedural knowledge for learners to monitor, adjust and control their learning. At the same time, feedback, in turn, enables learners to become self-regulated. Shepard (2005) advanced that formative feedback can develop students’ metacognition and self-reflection. In order to enforce learners’ metacognitive regulation, it is important for students to be able to adopt a standard for evaluation of learning. To obtain these goals, teachers need detailed data on learners’ behaviors and performance (automatically generated by the platform or some plug-ins for learners to consult and teachers to check) in online learning. At the same time, they bear the responsibilities of reviewing and reflecting on the data for developing learners’ metacognition, namely, self-awareness, self-evaluation and self- regulation.

## Conclusion

This research reveals that participants take positive attitudes toward feedback for its supervisional function and with a clear understanding of the relationship between online feedback and metacognition in which self-awareness, self-reflection, self-evaluation and self-regulation were emphasized. Nevertheless, second language learners misinterpreted self-consciousness about online feedback and admitted inappropriate knowledge of strategies and tasks that characterizes online L2 learning. Online feedback was primarily considered by them, as a reflection rather than as a process of regulation in online learning. Such a view of online feedback might have been shaped, complexly by the nature of online feedback, the comments they had received, and the online context in which they had been engaging with second language learning. In addition, it is suggested that students seem to be responsible for monitoring online feedback while implementing corresponding revision over online feedback. The importance of developing students’ metacognition in online feedback was emphasized as well.

This research certificated that metacognition in teacher feedback, as a component of autonomous learning, can support the development of learners’ autonomy in online L2 education. Since autonomous learning consists of cognition, metacognition, and motivation (O’Leary and Sloutsky, 2019), further researches can focus on the role of other two components, namely, cognition and/or motivation in empowering livestream teaching (see Maslow, 1943, 1987).

## Data Availability Statement

The raw data supporting the conclusions of this article will be made available by the authors, without undue reservation.

## Ethics Statement

Ethical review and approval was not required for the study on human participants in accordance with the local legislation and institutional requirements. The patients/participants provided their written informed consent to participate in this study.

## Author Contributions

FW framed the research. FM collected the data and drafted. SW, SL, and LP proofread. ZL supervised the research. All authors contributed to the article and approved the submitted version.

## Conflict of Interest

The authors declare that the research was conducted in the absence of any commercial or financial relationships that could be construed as a potential conflict of interest.

## Publisher’s Note

All claims expressed in this article are solely those of the authors and do not necessarily represent those of their affiliated organizations, or those of the publisher, the editors and the reviewers. Any product that may be evaluated in this article, or claim that may be made by its manufacturer, is not guaranteed or endorsed by the publisher.
